# Effect of Nano-Magnesium Oxide on the Expansion Performance and Hydration Process of Cement-Based Materials

**DOI:** 10.3390/ma14133766

**Published:** 2021-07-05

**Authors:** Yuxun Ye, Yanming Liu, Tao Shi, Zhuojun Hu, Lei Zhong, Haobo Wang, Yaohui Chen

**Affiliations:** 1 Department of Civil Engineering, College of Civil Engineering, Zhejiang University of Technology, Hangzhou 310023, China; 2111906127@zjut.edu.cn (Y.Y.); 1112006003@zjut.edu.cn (Y.L.); 2112006032@zjut.edu.cn (Z.H.); 2112006017@zjut.edu.cn (L.Z.); 2112006019@zjut.edu.cn (H.W.); 2112006009@zjut.edu.cn (Y.C.); 2 Department of Basic Education, Zhejiang University of Water Resources and Electric Power, Hangzhou 310018, China; 3 Zhejiang Provincial Key Laboratory of Engineering Structure and Disaster Prevention Technology, Hangzhou 310023, China

**Keywords:** nano-magnesium oxide (MgO), fly ash, expansion performance, hydration process, stability

## Abstract

Many scholars are concerned about the effect of nano-MgO as an expansion agent on the performance of cement-based materials at an early age, but over a long period less attention is paid to expansion stability and mechanical properties. This article examines the influence of nano-MgO on the long-term consistency, fluidity, expansion stability, hydration, and mechanical properties of 30% fly ash cement-based materials and improves research into nano-MgO as an expansion agent. Expansion performance, flexural and compressive strength, and stability after boiling and autoclave treatment were tested for specimens mixed with a 2, 4, 6, 8 and 10% cementitious material mass of nano-MgO. X-ray diffraction (XRD) and scanning electronic microscopy (SEM) were employed to study their hydration process and microstructure. The results showed that nano-MgO had an obvious effect on the consistency, fluidity and expansion performance of cement paste. After curing in water for 365 days and autoclaving thereafter, the hydration of nano-MgO was relatively complete. The volumetric expansion pressure of the magnesium hydroxide (Mg(OH)_2_) crystals and the crystallization pressure generated after their continuous precipitation were the main reasons for the expansion of the slurry. Nano-MgO improved the microstructure of cement paste and significantly enhanced its long-term flexural strength and compressive strength. When the content of nano-MgO was less than 10%, the cement with 30% fly ash had good long-term stability with the potential to compensate for the shrinkage of large-volume concrete.

## 1. Introduction

Portland cement, a widely used building material, cracks easily because of chemical, drying and autogenous shrinkage caused by temperature stress. These cracks reduce building safety, shorten service life, and cause huge losses to the economy and society. Micro cracks, invisible to the naked eye (less than 0.05 mm), allow acidic substances in the air to enter the concrete through pores, causing carbonation, corrosion and alkali aggregate reaction. Over time, this leads to cracking and spalling and then to steel corrosion. Moreover, carbonated concrete aggravates shrinkage deformation, leading to the development of macro cracks and a loss of strength, resulting in the destruction of the structure. To control concrete cracking and improve durability, expansion agents are often added to offset shrinkage. Lightly burned magnesium oxide (MgO) has a micro-expansion effect with relatively stable hydration products, compensates for the shrinkage of cement-based materials over the long term, and can also replace some cementitious materials. Therefore, in recent years, lightly burned MgO has gradually attracted the attention of scholars as a novel expansion agent. Pengkun Hou [1] found that ultrafine magnesium oxide contributed to an increase in compressive strength and a decrease in shrinkage of cement-based materials at the very early age but hindered hydration, coarsened the microstructure, and decreased later-age shrinkage to a much smaller extent than did lightly burnt magnesium oxide. According to Rıza Polat [2,3], nano-MgO decreased autogenous shrinkage, and setting time decreased with an increased in nano-MgO. The highest reduction was observed for mixes containing 7.5% CaO and nano-MgO and resulted in a reduction of autogenous shrinkage by 80% at 28 days [4]. White [5] studied the effect of a high-concentration of MgO clinker (MgO content 4.45–7.21%) on expansion. The results showed that, due to periclase hydration, the cement-based materials expanded by 0.15–0.20% in 1–5 years and became stable in about 4–6. In the 1970s, China first used cement with high MgO content (4.5% MgO in clinker) to build the Baishan Gravity Arch Dam in Jilin Province. Although no temperature control measures were taken during construction, only a few penetrating cracks were detected after several years [6]. Research over the past 40 years has shown that MgO can be used as an expansion component to compensate for the shrinkage of large-volume concrete in dams caused by falling temperatures. The expansion product of hydrated MgO, magnesium hydroxide (Mg(OH)_2_) crystals, is stable [6]. In the 1990s, the Three Gorges Dam was also constructed with cement containing a high MgO content. Due to the slow hydration of MgO in the clinker, thermal shrinkage of the concrete during the cooling stage was partially offset by delayed expansion [7]. Qing et al. found that with 4.5–5.0% MgO in the clinker and 2.8–3.4% SO_3_ in cement, ettringite and brucite expansion produced by periclase hydration in the cement paste had continuity, entirety, and stability [8].

The hydration rate and degree of MgO are critical for determining its expansion effect. The in situ growth and recrystallization of the Mg(OH)_2_ crystals increase the volume of the solid phase, which leads to MgO expansion. However, as the conversion of MgO to Mg(OH)_2_ produces a molar volume expansion of 117%, too high a MgO content in the hardened cement-based materials may lead to excessive expansions and even cracks. When the content of lightly burned MgO was less than 5%, the self-expansion rate of the concrete was lower than 120 mm/m per year [9]. Therefore, it is difficult to use the expansion effect of lightly burned MgO to completely compensate for the autogenous and temperature shrinkage. Due to the slow hydration of the heavily burned MgO in the early stage, the excessive expansion in the later stage leads to poor stability. Therefore, its content needs to be estimated and controlled during cement production and engineering applications. Also, the temperature sensitivity of cement and the MgO expansion agent in composite cementitious material is different. As MgO has a lower apparent activation energy and is more sensitive to temperature, the hydration process and mechanism of composite cementitious materials are more complicated.

With the rapid development of nanomaterial technology, several scholars have employed nanomaterials to improve the, impermeability and durability of cement-based materials. Liu et al. studied the basic properties and dispersion methods of carbon nanotubes (CNTs) and carbon nanofibers (CNFs) [10,11,12]. The results of the study showed adding CNTs significantly inhibited self-shrinkage, and the maximum shrinkage rate was more than 40%. Shi et al. found that in a slurry containing 0.06 wt% graphene, the drying shrinkage was inhibited by 27.4% [13]. Moradpour [14] studied the impact of nano-MgO and concluded that the expansion rate of Portland cement paste containing 3 and 5% of nano-MgO was 0.10 and 0.12%, respectively. Some scholars have found that nano-MgO reduced the fluidity of cement paste and delayed its initial and final setting timed [2,3,4]. Ye [15] and Kahidan concluded that the expansion capacity of cement paste mixed with nano-MgO increased after curing in water at 20 °C for 365 days. The agglomeration of nano-MgO causes its hydration to form a protective layer to prevent water from entering the agglomerates. Therefore, the cement paste still had room to expand after autoclaving, and the expansion increment could reach 70%. Qureshi [16] improved the healing of drying shrinkage cracks in conventional silicate cements by adding an expansion mineral such as activated magnesium oxide (MgO). Test results showed that cracks up to 500 μm were sealed in most of the MgO-containing samples after 28 days. In microstructural investigations, highly expansive Mg-rich hydro-carbonate bridges were found along with traditional calcium-based, self-healing compounds (calcite, portlandite, calcium silicate hydrates and ettringite). Qureshi [17] studied the impact of expansive minerals, namely magnesium oxide, bentonite clay, and quicklime on the early-age autogenous self-healing capacity of Portland cement (PC) paste. He found that cracks in the range of 180 µm healed efficiently in a mineral containing mixes within 28 days. Litina and Bumanis et al. found that the healing obtained by adding MgO-based expansive agents was comparable and complementary to the reference specimens but showed greater efficiency in sustaining long-term healing and later-age crack healing because the active agents remain unreacted for longer in the matrix [18].

Although several works have been performed using nano-MgO as an expansion agent, the mixed content of nano-MgO and the hydration of nano-MgO-cement are debated. Limited work has been done on the long-term expansion stability of cement-based materials; therefore, the mixed content of nano-MgO and the hydration process of nano-MgO-cement was studied in this work, with special attention to long-term expansion stability. In addition, it will provide a solid theoretical foundation and practical direction for the optimal design and application of nano-MgO to compensate for shrinkage in large-volume concrete.

## 2. Test Materials and Methods

### 2.1. Test Materials

**Figure 1:**
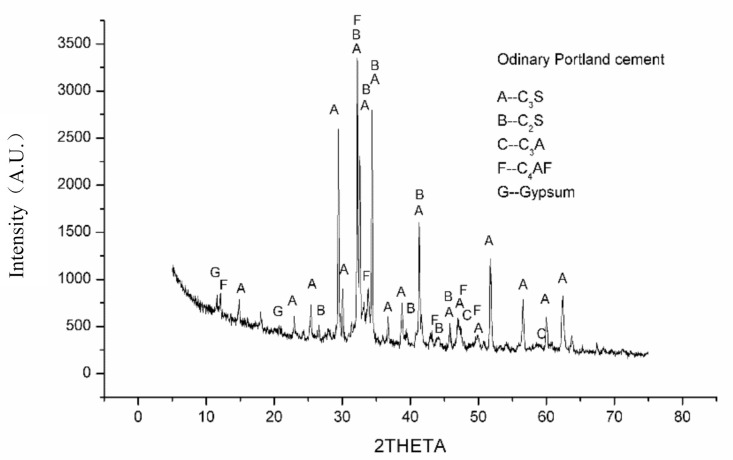
XRD pattern of ordinary Portland cement.

This study used Grade 42.5 ordinary Portland cement containing 80% clinker, 5% gypsum, and 15% mineral additives. Its specific surface area was 330 m^2^/kg, while the 28 days compressive strength and flexural strength were 47.9 MPa and 7.6 MPa, respectively. Its chemical composition is listed in Table 1 and its X-ray diffraction (XRD)pattern is shown in Figure 1. Figure 1 showed that the cement mainly contained C_3_S, C_2_S, C_3_A and C_4_AF as well as a small amount of gypsum.

**Figure 2:**
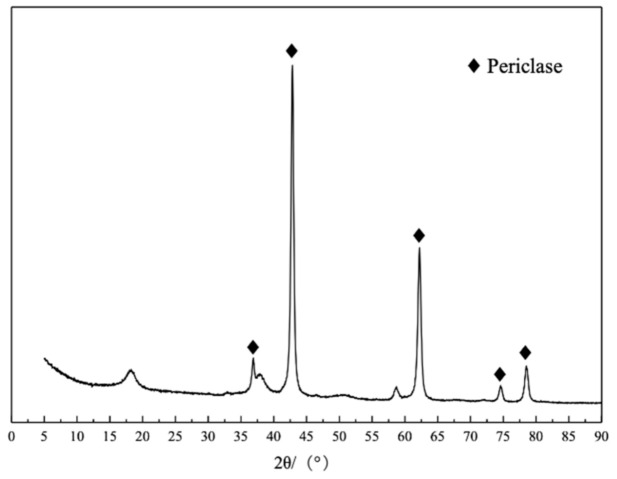
XRD pattern of nano-MgO.

**Figure 3:**
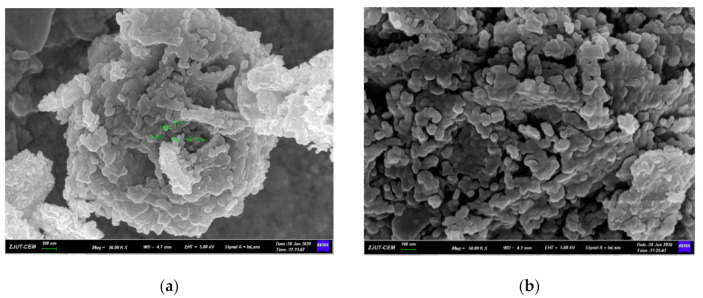
SEM images of nano-MgO. (**a**) with dimensioning and (**b**) without dimensioning.

Nano-MgO was purchased from Hangzhou Wanjing New Materials Co., Ltd., Hangzhou, China, with a specific surface area of 30–50 m^2^/g and an average particle size of 50 nm. Its chemical composition, XRD pattern, and microstructure and morphological characteristics are presented in Table 1, Figure 2 and Figure 3, respectively. The XRD pattern showed that periclase was the main mineral of nano-MgO. Figure 3a showed the labeled spherical nano-MgO with a diameter of 40.75 nm; Figure 3b showed uniform spherical nano-MgO agglomerated and stacked with each other.

**Figure 4:**
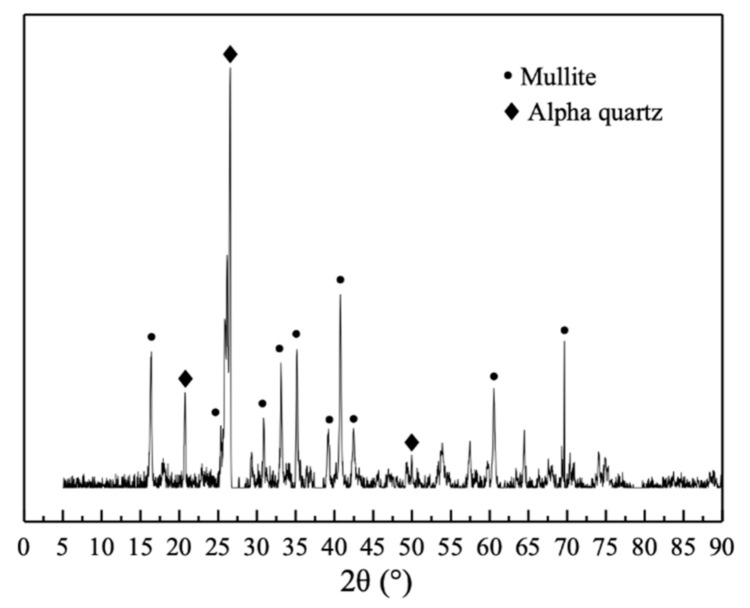
XRD pattern of fly ash.

Used F·II type fly ash was purchased from Ningbo Zhenhai Power Plant in Ningbo, China. Its chemical composition and XRD pattern are displayed in Table 1 and Figure 4, respectively. The XRD pattern revealed that mullite and quartz exhibited very sharp diffraction peaks, while the diffraction peaks of other crystalline substances were fainter. Chinese ISO standard sand and tap water were used for all the tests.

### 2.2. Test Methods

#### 2.2.1. Consistency and Fluidity of Cement Paste

Based on the mix ratio provided in Table 2, the test method of cement paste consistency was carried out in accordance with GB/T1346-2011, “Test Methods for Water Requirement of Normal Consistency, Setting Time and Soundness of the Portland Cement”. As cement paste mixed with nano-MgO showed poor fluidity, a direct measurement could not give accurate results. Therefore, thew GB/T2419-2005, “Test Method for Fluidity of Cement Mortar”, was used.

#### 2.2.2. Expansion Performance Tests of Cement Paste

The control sample was composed of 70% ordinary Portland cement and 30% fly ash. Under dry conditions, nano-MgO and fly ash were mixed into the cement to prepare the samples for experiments. The water-binder ratio of all the samples was 0.28. The mixing ratios of the samples are shown in Table 2. Three samples were used for each mix ratio group. The sample size was 25 mm× 25 mm× 280 mm. After water curing, the expansion rates of the samples were tested after 2, 3, 7, 14, 28, 120, 180, and 365 days and autoclave curing. The linear expansion tests of the cement paste were conducted according to JC 313-2009 “The Test Method for the Linear Expansion of Cement Paste”. The expansion-rate test was calculated according to Formula (1).

(1) $$E_{x}=\frac{L_{x}-L_{1}}{250}\times 100$$

In the above formula, *E_x_* indicates the expansion rate of the specimen at a certain age in percent (%); *L_x_* denotes the length reading (mm) of the specimen at a certain age in; *L_x_* shows the initial length (mm) reading of the piece, and 250 means that the effective length of the specimen was 250 mm.

#### 2.2.3. Strength Tests of Mortar

The samples of 70% Portland cement with nano-MgO and 30% fly ash was thoroughly mixed under dry conditions. The mixing ratios of the samples are listed in Table 3. Three samples were used for each set of mix ratios. The size of the sample was 40 mm× 40 mm× 160 mm. The specimens were cured for 28 and 365 days, autoclave cured, and then their compressive strength and flexural strength were measured. The autoclave curing was performed by curing the sample in a 216 °C and 2.0 MPa saturated steam for 6.0 h after curing in water at 20 °C for 365 days. The model of the autoclave was YZF-2A, which has an inner diameter of 160 mm, volume of 0.0085 m^3^, and maximum allowable steam pressure of 25 MPa. The flexural and compressive strengths of the specimens were tested using a compression bending machine and a universal testing machine, respectively. The loading speed of the compressive strength test was controlled at 2.4 ± 0.2 kN/s, and the compressed area of the specimens was 1600 mm^2^. The loading speed of the flexural strength test was automatically controlled at 50 N/s. The specimens used for the compressive strength test were adopted after the flexural strength test. The strength of each group of specimens was taken as the arithmetic mean of the measured values of the three specimens.

#### 2.2.4. Stability Tests of Cement Paste

The stability tests of cement paste included a boiling and an autoclave test. The expansion rates of the test pieces after boiling were measured according to GB/T1346-2011 “Test Methods for Water Requirement of Normal Consistency, Setting Time and Soundness of the Portland Cement”. Then, per GB/T 750-1992 “Autoclave Method for Soundness of Portland Cement”, the expansion rates of the specimens after autoclaving were measured. Each sample was formed with two test pieces. After the specimens were cured, the distance to the tip of the pointer (A) was measured with the Ray’s clamp tester. Then the specimens were put into the boiling box and heated to boiling within 30 ± 5 min and kept boiling for 180 ± 5 min. The distance to the tip of the pointer of the Ray’s clamp was measured after boiling (C). When the average value of the increased distance of the specimens (C–A) was not greater than 5.0 mm, the cement stability was considered to be qualified. Notably, the initial expansion value of the hardened slurry was set to 0 at 1 d, and the expansion amount after both boiling and autoclaving was compared with the initial expansion value. The amount of expansion during boiling was that of the expansion after the boiling treatment, while the amount of expansion during autoclaving was the difference between the amount of expansion after autoclaving and after boiling. The amount of expansion after boiling and autoclaving was the final amount of expansion, which was equal to the sum of the expansions during boiling and autoclaving. Ordinary Portland cement replaced with 30% fly ash can be considered to be a composite Portland cement. When the expansion rate of the sample after autoclaving was not more than 5000 μm/m, it was considered to have qualified test stability.

#### 2.2.5. X-ray Diffraction (XRD) Tests

An X-ray diffractometer (X’Pert PRO, PNAlytical, Almelo, Overijssel, The Netherlands), with a Cu target and wavelength of Cu Kα (0.1541 nm) was employed. The scanning angle range was set to 2θ = 5–80°, and the step was 0.033°. Before the tests, the samples were placed in absolute ethanol for a predetermined age to stop hydration, and then dried in a vacuum drying oven at 60 °C for 8 h. The dried sample was ground in an agate mortar, processed with a 200-mesh standard sieve, and then sent for testing.

#### 2.2.6. Scanning Electronic Microscopy (SEM) Tests

A scanning electron microscope (S-4700 (II), Hitachi, Chiyoda District, Tokyo, Japan) cold field emission scanning electron microscope) was employed for observations. The secondary electron image resolution was 15 kV: ≤1.5 nm, 1 kV: ≤2.1 nm. The magnification was about 50–100 k times and the acceleration voltage was 0.5–30 kV. Before the tests, the samples were placed in absolute ethanol for a predetermined period time to stop hydration, and then, dried in a vacuum drying oven at 60 °C for 24 h. After the sample was dried, it was sent for testing immediately to prevent it from being carbonated by the air.

## 3. Test Results and Discussion

### 3.1. Effect of Nano-MgO Content on the Consistency and Fluidity of Cement Paste

**Figure 5:**
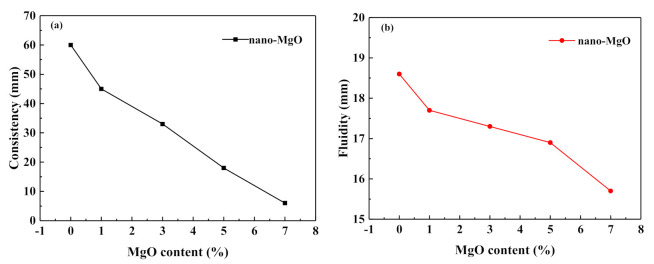
Influence of MgO on the consistency (**a**) and fluidity (**b**) of cement paste.

Figure 5a showed that the effect of nano-MgO on the consistency of cement paste was very significant. When the amount of nano-MgO mixed gradually increased, the consistency value of the specimen gradually decreased. When the dosage of nano-MgO was 1%, the sinking depth of the test cone was 45 mm. when the dosage was 3%, the consistency of the specimen was 33 mm. When the dosage was more than 3%, the consistency value of the specimen decreased more obviously. Figure 5b illustrates that the effect of nano-MgO on the fluidity of cement paste was also very significant. With the increase of nano-MgO dosage, it decreased almost linearly, which was consistent with the law of cement paste consistency. Compared with the control group, the fluidity of the specimen was 11.4 mm when the dosage of nano-MgO was 7%, which was 38.7% less than in the control group. Xiaoyan Wang [19] investigated the effect of nano-MgO on the fluidity of oil-well cement paste and found a similar phenomenon. The effect of nano-MgO dosage on the fluidity of cement paste was relatively obvious, and the fluidity of cement paste decreased with the increase in nano-MgO dosage. When the nano-MgO dose was 1.0%, the fluidity of the oil-well cement slurry worsened because the water demand of nano-MgO increased and generated Mg(OH)_2_, which also caused the consistency of the cement paste to become worse.

The nano-MgO has a small bulk density, which may affect the bulk density of the freshly mixed specimens. Therefore, it may disrupt the uniform particle assembly. In addition, it has a large specific surface area and high hydration activity, so it reacts with water rapidly. In fresh cement paste, nano-MgO consumed more free water for hydration and covering its surface layer [20,21,22], thus reducing the water–cement ratio and leading to reduced fluidity and consistency. Xue Zhang found that when a high MgO admixture of cement was used or excess MgO was used to mix high performance concrete, a higher water–solid ratio was required [23].

### 3.2. Effect of Nano-MgO Content on Expansion Performance of Cement Paste

Nano-MgO doping can significantly improve the expansion performance of cement paste. As its content rises, expansion performance increases and is amplified with the extension of the curing age. At an early age, the expansion of the cement paste sample mixed with nano-MgO was significantly higher than that of the control group. This may be because the nano-MgO particles were small and Mg(OH)_2_ crystals were quickly formed due to hydration. On the one hand, Mg(OH)_2_ crystals grow on the walls of the pores around and near the MgO particles, and the increased volume of Mg(OH)_2_ crystals squeezes the surrounding cement paste and causes expansion [24,25,26]. On the other hand, Mg(OH)_2_ crystals continuously separate from the supersaturated solution and continue to grow in the original position, generating crystallization pressure on the surrounding slurry it to expand [27].

**Figure 6:**
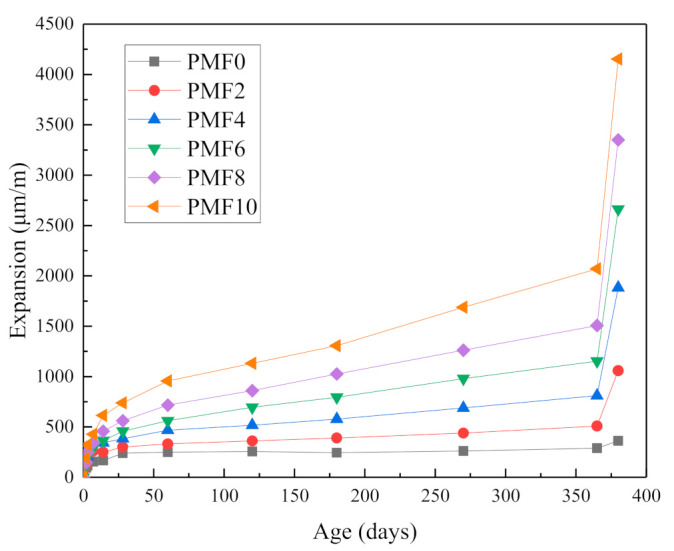
Effect of nano-MgO content on expansion performance of cement paste.

Figure 6 shows that after autoclaving, the expansion of all the samples increased significantly with the rise in nano-MgO. The expansion of the sample without nano-MgO was 363 μm/m after autoclave curing, while the expansion amounts were 1060, 1883, 2663, 3350, and 4152 μm/m when nano-MgO was 2, 4, 6, 8, and 10%, respectively, after autoclave treatment. Compared with the samples without nano-MgO, the expansion of the above samples increased by 192, 419, 633, 823, and 1044%, respectively. The XRD tests demonstrate that MgO in the samples was reduced and largely hydrated. This may have been due to the sufficient hydration of MgO in the samples under the autoclave environment, and rapid Mg(OH)_2_ accumulation on the surface of MgO, which expanded to relatively high values. Therefore, after autoclave treatment, the expansion of all the samples increased greatly. The above experimental results are consistent with the literature [28]. In most cases, the literature revealed very high expansion for autoclaved bar specimens made of cement pastes without the addition of fly ash. Sherir et al. [29] stated that the autoclave test rapidly accelerates the formation of Mg(OH)_2_ crystals and the hydration of C–S–H as well. Therefore, weaker microstructures might be produced in autoclave curing due to considerable expansive stresses created on the surrounding pores when Mg(OH)_2_ crystals are formed.

### 3.3. Effect of Nano-MgO Content on Mortar Strength

**Figure 7:**
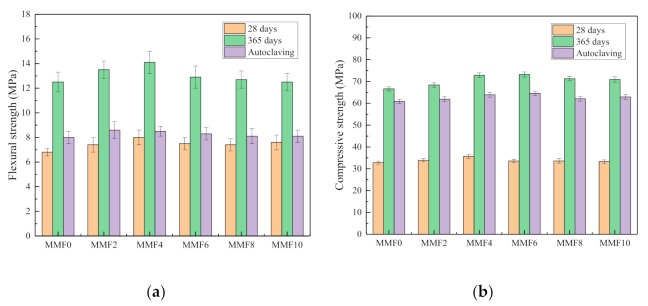
Effect of nano-MgO content on mortar strength. (**a**) flexural strength and (**b**) compressive strength.

Figure 7 displays the related data of mortar strength with different nano-MgO content. Compared with the blank sample, the flexural strength and compressive strength of all the samples mixed with nano-MgO at 28 days significantly increased. Nano-MgO improved the 28 days strength of the sample, and the flexural and compressive strengths increased at most by 17.6 and 8.8%, respectively, when the content of nano-MgO was 4%. Both Hou [1] and Ye [15] also concluded that nano-MgO increased the 28 days compressive strength, probably because the microstructure improved after nano-MgO incorporation. With the extension of the curing age, the flexural and compressive strengths of all the samples gradually increased, most likely because fly ash underwent a pozzolanic reaction, which improved the interface transition zone of the cement stone [30]. At 28 and 365 days, compared with the blank sample, the flexural and compressive strength in all samples did not increase significantly with increased nano-MgO content, but after autoclaving were significantly reduced after 365 days. When the nano-MgO content was 4%, the decrease in these strengths was 39.7 and 12.3%, respectively. This may be because the microstructure of the cement stone was destroyed during high-temperature autoclaving, which caused micro cracks to continue developing, thereby deteriorating the mechanical properties of the mortar [29]. In addition, C–S–H hydration was expedited, leading to the loss of cohesive forces within the cement paste. After the addition of fly ash to the paste, pozzolanic reaction occurred forming strong C–S–H products in the surrounding microstructures and limiting the formation of Mg(OH)_2_ crystals [31,32,33]. Compared with the blank sample, the flexural and compressive strengths of the nano-MgO sample did not decrease significantly. In summary, the mixed content of nano-MgO had no significant effect on the strength of cement mortar and did not degrade its mechanical properties.

### 3.4. Relationship between Stability of Cement Paste and Nano-MgO Mixed Content

**Figure 8:**
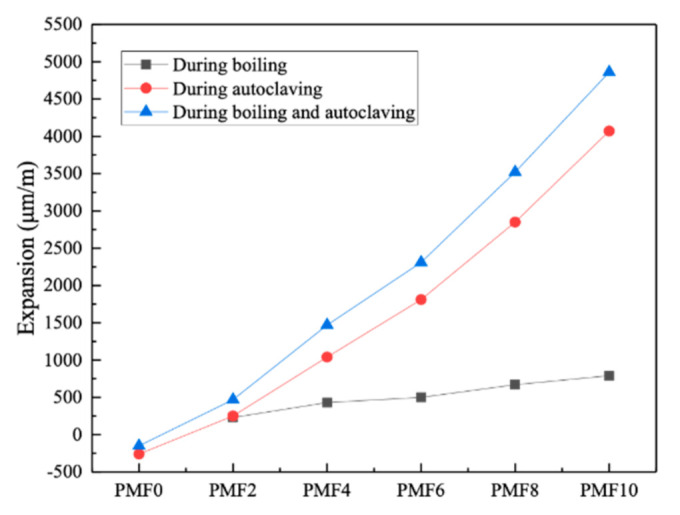
Effect of nano-MgO content on stability of cement paste.

Figure 8 shows that an appropriate mixed amount of nano-MgO significantly improved the expansion performance of the cement paste after boiling and autoclaving, which reflected the paste’s stability. Nano-MgO reacted slowly with water, but it had a very fine particle size and high surface area [3,34], properties that increased its reaction and made for a more uniform expansion in the cement paste [2]. Its expansion after boiling and autoclaving was amplified with an increased amount of nano-MgO because the MgO hydration rate was greatly affected by temperature. Compared with the boiling treatment, the autoclave curing further improved the activity of MgO, and the increased hydration rate of MgO led to volume expansion of slurry. When the content of nano-MgO did not exceed 10%, the expansion rates of all the samples after boiling and autoclave treatment were less than 5000 μm/m; hence, the stability of the cement paste was confirmed. Wang drew a similar conclusion when she found that the volume of cement with 30% fly ash admixture swelled stably when the nano-MgO admixture did not exceed 10%. The specimens had a stability consistent with the objectives of the study [29].

### 3.5. Analysis of XRD Test Results

**Figure 9:**
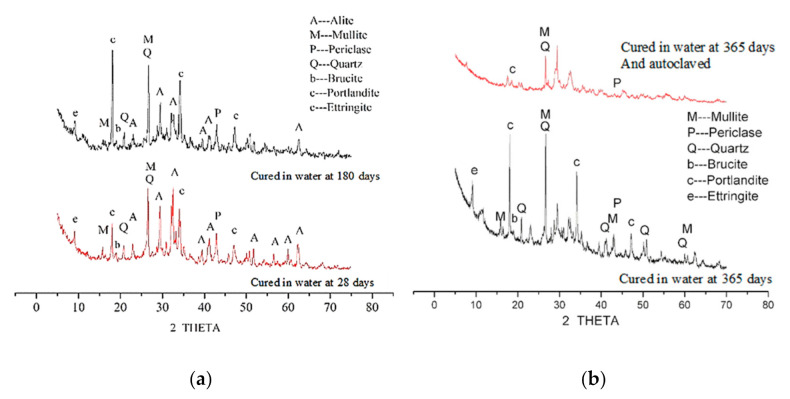
XRD patterns of hydration of cement paste mixed with 6% nano-MgO (28, 180, and 365 days). (**a**) cured in water at 28 days and 180 days and (**b**) cured in water at 365 days, and cured in water at 365 days and autoclaved.

As shown in Figure 9, XRD patterns were used to determine the change of nano-MgO hydration with time. With the extension of the curing age in water, the diffraction peak of periclase gradually decreased, while the diffraction peak of brucite gradually increased, indicating that nano-MgO was being continuously hydrated to form Mg(OH)_2_ crystals. According to Deng et al. [35,36], MgO was hydrated in a solution of higher pH, and from this it could be inferred that because the nano-MgO particles were very fine and mixed homogeneously within the cement tiny crystals of Mg(OH)_2_ might precipitate onto the surface of the cement particles to form a “protective layer” [3,4]. In addition, the diffraction peak of ettringite gradually increased, and the diffraction peak of mullite gradually decreased, indicating that fly ash was gradually consumed and the degree of cement hydration continuously increased. The XRD pattern s of the sample that had been cured for 365 days in water and then autoclaved showed that the diffraction peak intensity of periclase was extremely weak, whereas those of mullite and quartz decreased significantly. This indicated that autoclave treatment significantly promoted the rapid hydration of nano-MgO [15,25], which increased the expansion rate of the cement stone.

### 3.6. SEM Analysis

**Figure 10:**
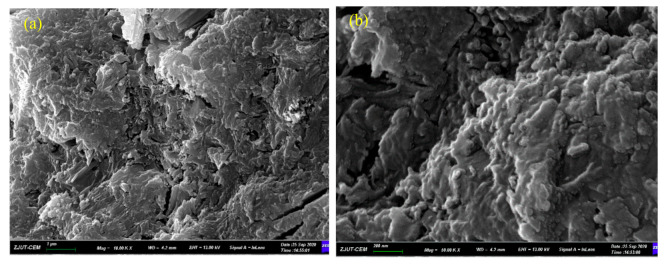

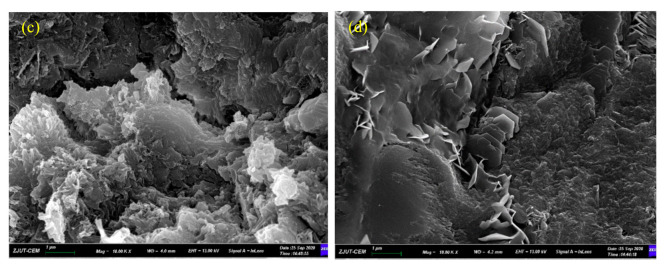
Scanning electron microscope images of 28 days sample. (**a**,**b**) Samples without nano-MgO; (**c**,**d**) Samples mixed with 6% nano-MgO.

Figure 10 displays the scanning electron micrograph of 28 days nano-MgO cement paste under standard curing conditions at 20 °C. Compared with the blank group, the samples mixed with 6% nano-MgO demonstrated prominent wide cracks. The XRD tests show that MgO was continuously hydrated to produce Mg(OH)_2_. The volume expansion of MgO particles produced expansion stress on the surrounding cement paste; hence, the cement stone developed micro-cracks. Mo found a similar phenomenon in which the particles of the MgO expansive agent were distributed in the cement pastes, and each particle hydrated locally [37]. The hydration of MgO induced self-expansion of the MgO expansive agent particle in a confined space surrounded by the hydrated cement matrix, and the confined expansion produced expansive stress, which eventually expanded the hydrated cement matrix [4]. However, the blank samples were dense, which may have been due to the slow hydration of fly ash during the curing process and the generated hydrated calcium silicate gel filling the pores of the slurry. The sample mixed with 6% nano-MgO displayed hexagonal plate-shaped Ca(OH)_2_ and flake-shaped Mg(OH)_2_ stacked and agglomerated with each other, indicating that the sample had been hydrated gradually, which was consistent with the experimental results in reference [38]. Peng Liu studied the development of expansion stresses, brucite formation speed, and the morphology of the brucite [39]. As the MgO expansive agent became hydrated and hydromagnesite rapidly filled the pores of the compacted body, a larger number of hexagonal plates of brucite together formed a dense structure. The Mg(OH)_2_ crystals overlapped into a scaffold shape, recrystallized in the solution and gradually grew, thereby squeezing the slurry or with the Ca(OH)_2_ crystals causing expansion.

Mo et al. [4] assessed the morphology of cement pastes produced with MgO water-cured for 270 days at 40 °C. Due to the influence of alkali on MgO hydration, the hydration products in Mg(OH) _2_ were smaller and more irregular compared to the reference paste without additional MgO. In addition, cracks were observed at the sintered MgO particle interface, associated with its expansion at the particle boundary [40]. Ye Qing et al. investigated the cement paste mixed with 6% nano MgO cured at 40 °C for 365 days [25]. It was found that the magnesite diffraction peak was still present, but the peak height was very low.

## 4. Conclusions

The effect of nano-MgO on the expansion performance of cement-based materials replaced with 30% fly ash was investigated. Their hydration was studied with special attention to the long-term expansion stability of cement-based materials. The following conclusions were drawn:

(1) The effect of nano-MgO on the consistency and fluidity of cement paste is obvious. When the mixing amount of nano-MgO gradually increases, the consistency value of the specimen’s fluidity gradually decreases. When the amount exceeds 3%, the consistency value of the specimen decreases more obviously.

(2) Compared with the samples without Nano-MgO, when the Nano-MgO content was 2, 4, 6, 8 and 10%, the expansion of the samples after autoclave treatment increased by 192, 419, 633, 823, and 1044%, respectively. The volumetric expansion pressure of Mg(OH)_2_ crystals and the crystallization pressure generated after their continuous precipitation were the main reasons for the expansion of slurry.

(3) Nano-MgO improved the microstructure of cement stone, resulting in a significant increase in flexural and compressive strength. Autoclave treatment could destroy the microstructure of the cement stone, promote the development of microcracks and degrading the mechanical properties of the mortar.

(4) The XRD tests shows that after curing in water for 365 days and autoclaving, the diffraction peak intensity of periclase was already feeble, the diffraction peak intensity of brucite is large, and the hydration of nano-MgO is relatively complete.

(5) The SEM analysis demonstrated that the hexagonal plate-shaped Ca(OH)_2_ and the stacked and agglomerated flake Mg(OH)_2_ squeezed the slurry or squeezed with other Mg(OH)_2_ crystals, causing the slurry to expand.

(6) When the content of nano-MgO did not exceed 10%, the cement with 30% fly ash displayed good stability and had the potential to compensate for the shrinkage of large-volume concrete.

## Figures and Tables

**Table 1:** Chemical compositions (wt%) of ordinary Portland cement, fly-ash, and nano-MgO.

Material	CaO	SiO_2_	Al_2_O_3_	Fe_2_O_3_	MgO	SO_3_	K_2_O	Na_2_O
OP cement	59.53	21.63	5.85	4.90	2.02	2.80	0.65	0.23
Fly-ash	6.06	52.34	31.74	4.26	1.66	0.40	0.95	0.81
Nano-MgO	-	-	-	-	99.90	-	-	-

**Table 2:** Doping ratio of cement paste.

Paste Code	Doping Proportion of Paste (g)			
	OP Cement	Fly-Ash	Nano-MgO	Water
PMF0	350	150	0	140
PMF2	343	147	10	140
PMF4	336	144	20	140
PMF6	329	141	30	140
PMF8	322	138	40	140
PMF10	315	135	50	140

**Table 3:** Doping ratio of cement mortar.

Mortar Code	Doping Proportion of Mortar (g)				
	OP Cement	Fly-Ash	Nano-MgO	Water	ISO Sand
MMF0	315	135	0	225	1350
MMF2	309	132	9	225	1350
MMF4	302	130	18	225	1350
MMF6	296	127	27	225	1350
MMF8	290	124	36	225	1350
MMF10	284	121	45	225	1350

## Data Availability

The data presented in this study are available upon request from the corresponding author.
